# Vitrectomy for ocular toxoplasmosis: a case report and literature review

**DOI:** 10.3389/fmed.2026.1888978

**Published:** 2026-07-08

**Authors:** Xiaomin Zhang, Hongbo Huang, Limei He, Heruo Wei, Lirong Wei, Jianwei Zhai, Huanyan Wang, Guangjie Han

**Affiliations:** Department of Ophthalmology, Liuzhou Red Cross Hospital, Eye Hospital of Liuzhou City, Liuzhou, Guangxi, China

**Keywords:** Goldmann-Witmer coefficient, ocular toxoplasmosis, polymerase chain reaction, uveitis, vitrectomy

## Abstract

Ocular toxoplasmosis (OT) is a parasitic ocular disorder triggered by *Toxoplasma gondii* infection, ranking as a leading cause of posterior uveitis. This pathogen spreads globally, with nearly one-third of the world’s population having previous infection evidence, and OT may cause irreversible severe visual loss; its typical fundus features include vitreous opacities and focal yellowish-white inflammatory chorioretinal lesions, while active lesions often emerge beside old pigmented chorioretinal scars, and lesions involving the macula or optic disc usually lead to poor visual outcomes. Human immune function changes with age: it is immature in infancy, matures in young adulthood, and declines in the elderly via immunosenescence, which weakens innate and adaptive immunity, induces chronic low-grade inflammation, and raises infection risk. OT diagnosis mainly relies on typical fundus manifestations, while atypical cases require combined serological detection, Goldmann-Witmer coefficient detection and intraocular fluid PCR tests. The classic triple therapy carries obvious adverse reactions damaging bone marrow, liver and kidneys, so trimethoprim-sulfamethoxazole serves as a common alternative anti-parasite drug, with corticosteroids added as needed to suppress inflammation. For severe complications such as dense vitreous opacification, retinal detachment, retinal breaks and vitreous hemorrhage, pars plana vitrectomy combined with intraoperative laser photocoagulation is the primary surgical option. This paper reports an immunocompetent elderly OT patient who achieved a favorable prognosis after combined surgical intervention and postoperative anti-Toxoplasma medication, and further summarizes the core diagnostic essentials of OT as well as the clinical indications and value of vitrectomy.

## Introduction

Ocular toxoplasmosis (OT) is a parasitic eye disease caused by infection with *Toxoplasma gondii* and is one of the major causes of posterior uveitis. *Toxoplasma gondii* is widely distributed worldwide, and it is estimated that approximately one-third of the global population has serological evidence of past infection (1–4). Ocular involvement can lead to severe visual impairment. The typical fundus manifestations of OT include vitreous opacities with focal yellowish-white inflammatory fundus lesions, often accompanied by uveitis. Active lesions may also arise adjacent to old pigmented chorioretinal scars. When the lesion involves the macula or optic disc, the visual prognosis is often poor (5–9). Immune function changes dynamically with age. The immune system is relatively immature in early life, matures during childhood and adulthood, and gradually declines in older age, a process known as immunosenescence. Age-related immune decline is characterized by impaired innate and adaptive immune responses, decreased vaccine responsiveness, and chronic low-grade inflammation, which may increase susceptibility to infections and other age-related diseases (10–12). The diagnosis of OT is based primarily on characteristic fundus findings. In atypical cases, laboratory tests such as serological testing, Goldmann-Witmer coefficient determination, and polymerase chain reaction (PCR) analysis of intraocular fluids should be combined for comprehensive judgment (13–19). Regarding treatment, the classic regimen of pyrimethamine, sulfadiazine, and folinic acid, with corticosteroids added when necessary although considered a standard regimen, is associated with significant side effects including bone marrow suppression, allergic reactions, and hepatorenal impairment, and lacks optimal practicality. Currently, trimethoprim-sulfamethoxazole is frequently used as alternative therapy in clinical practice, combined with corticosteroids when necessary to control inflammation. For serious complications, such as severe vitreous opacity, tractional or rhegmatogenous retinal detachment, retinal tears, or vitreous hemorrhage, pars plana vitrectomy, either alone or combined with cataract surgery, may be required as the preferred surgical intervention. Intraoperative laser photocoagulation and pharmacological treatment may also be performed as appropriate, as these approaches allow further management of fundus lesions (20–23). This article reports a typical case of ocular toxoplasmosis in an elderly patient without known immunodeficiency disease, who achieved a favorable outcome following vitrectomy combined with intraoperative laser photocoagulation and postoperative anti-Toxoplasma medication. In addition, it discusses the key diagnostic points of ocular toxoplasmosis and the indications and value of vitrectomy based on a review of the relevant literature.

## Case report

### Patient history

A 77-year-old female presented to our hospital with a complaint of “sudden vision loss in the left eye for more than 20 days.” Her past medical history included coronary heart disease, and she had undergone coronary stent implantation at Liuzhou Railway Central Hospital. She was currently taking oral atorvastatin calcium, clopidogrel bisulfate, metoprolol succinate sustained-release, and spironolactone tablets on a regular basis. She also had a history of surgery under general anesthesia for left knee disease and reported recent abdominal discomfort. Her neighbor kept cats, but the patient denied any history of close contact.

### Examination findings

**Figure 1:**
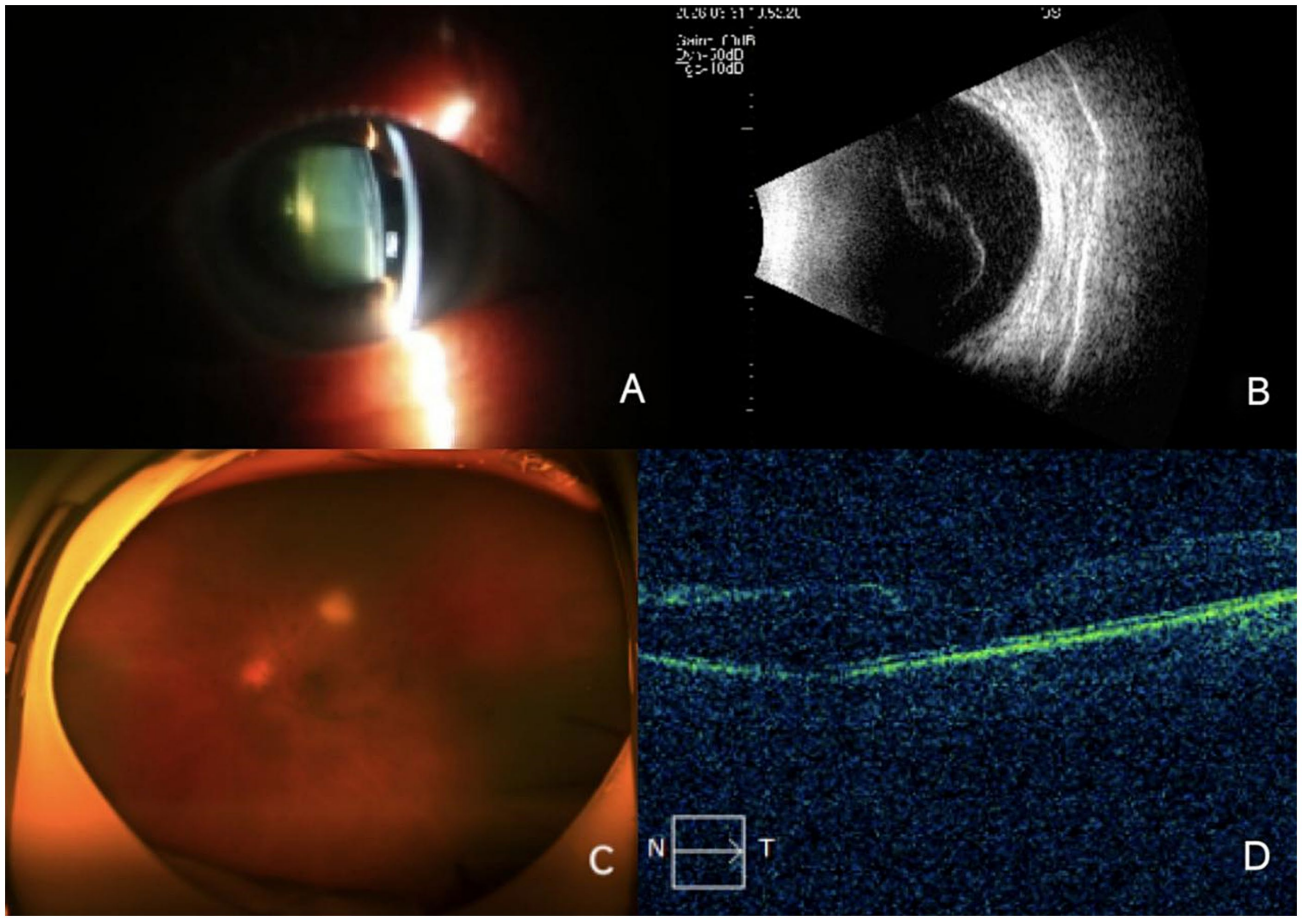
Pre-treatment images of the affected left eye. **(A)** Anterior segment photography showed marked lens opacity. **(B)** Pretreatment ocular B-scan ultrasonography revealed scattered vitreous opacities within the vitreous cavity. **(C)** Scanning laser ophthalmoscopy (SLO) showed inflammatory vitreous haze in the left eye, with an indistinct yellowish-white triangular lesion approximately 1 disc diameter in size at the superior vascular arcade; the retinal image quality was poor. **(D)** OCT showed an irregular retinal pigment epithelium layer in the macular region, macular edema, and poor signal quality.

Scanning laser ophthalmoscopy (SLO) and optical coherence tomography (OCT) examination results are shown in Figure 1. The ophthalmic examination findings are summarized in Table 1.

**Table 1:** Ophthalmic examination findings.

Examination item	Right eye (OD)	Left eye (OS)
Visual acuity (VA)	0.15, no improvement with correction	0.12, no improvement with correction
Intraocular pressure (IOP)	12 mmHg	14 mmHg
Eyelids	No eyelid erythema or edema, no entropion, ectropion, trichiasis, or ptosis	No eyelid erythema or edema, no entropion, ectropion, trichiasis, or ptosis
Lacrimal apparatus	Lacrimal punctum in normal position, no discharge on lacrimal sac compression	Lacrimal punctum in normal position, no discharge on lacrimal sac compression
Conjunctiva	No conjunctival hyperemia	No conjunctival hyperemia
Sclera	No scleral injection	No scleral icterus, ciliary injection present
Cornea	Clear	Clear
Anterior chamber	Axial depth approximately 3 corneal thicknesses, aqueous humor clear	Keratic precipitates (KP) positive, axial depth approximately 3 corneal thicknesses, aqueous cells and aqueous flare present
Iris	Iris texture clear	Iris texture slightly indistinct
Pupil	Round, approximately 3 × 3 mm, light reflex slightly sluggish	Round, approximately 3 × 3 mm, light reflex slightly sluggish
Lens	Lens opacity	Lens opacity
Vitreous	Vitreous opacity	Vitreous opacity
Fundus	Retina faintly visible and attached	An indistinct, yellow-white lesion of approximately 1 disc diameter was observed superior to the macula, the remaining retina was attached

Test results helpful for diagnosis and differential diagnosis are summarized in Table 2.

**Table 2:** Laboratory test results.

Laboratory inspection items	Results
Erythrocyte sedimentation rate (ESR)	49.00 mm/h (0–30)
High-sensitivity C-reactive protein (hs-CRP)	18.10 mg/L (0–10)
Cytomegalovirus IgG antibody (CMV-IgG)	>1000.00 AU/mL, positive
Herpes simplex virus type 1 IgG antibody (HSV-1-IgG)	219.26 AU/mL, positive
Quantitative rubella virus IgG antibody (RV-IgG)	23.62 IU/mL, positive
Serum *Toxoplasma gondii* IgG	7083.30 IU/mL (0–20)
Serum *Toxoplasma gondii* IgM	<1.00 AU/mL, negative
Serum anti-*Toxocara* IgG	9.60 U (0–9)
Intraocular fluid *Toxoplasma gondii* DNA	positive
Intraocular fluid anti-*Toxoplasma gondii* IgG	92.40 IU/mL (0–4)
Toxoplasma-specific Goldmann-Witmer Coefficient (GWC)	0.01 (0–2)

### Surgical procedure and postoperative treatment

On April 1, 2026, the patient underwent left-eye pars plana vitrectomy combined with phacoemulsification cataract extraction, primary intraocular lens implantation, intravitreal injection of triamcinolone acetonide 40 mg/mL, 0.01 mL, and laser photocoagulation of the retinal lesion. During the surgery, vitreous fluid was aspirated for intraocular fluid testing. The results are summarized in Table 2: serum *Toxocara* IgG: 9.6 U (elevated), serum *Toxoplasma* IgG: 7083.3 IU/mL (elevated), intraocular fluid *Toxoplasma gondii* DNA: positive, intraocular fluid anti-*Toxoplasma* IgG: 92.4 IU/mL (elevated), and a Toxoplasma Goldmann-Witmer coefficient of 0.01. Intraoperative findings included grade 2+ inflammatory vitreous haze in the left eye, a triangular lesion approximately 1 disc diameter in size at the superior vascular arcade, shallow retinal detachment surrounding the lesion, and retinal artery sheathing. Diagnosis: left eye ocular toxoplasmosis, left eye retinal arteritis (Table 3).

**Table 3:** NEI/Nussenblatt vitreous haze grading scale.

Grading	Criteria
0	No vitreous haze
0.5+/trace	Slight blurring of the optic disc margin
1+	Obscured view, but the optic nerve head and retinal vessels remain identifiable
2+	Obscured view, but retinal vessels remain identifiable
3+	Optic nerve head can be visualized, but the borders are blurry
4+	Optic nerve head/fundus view is obscured

Oral compound sulfamethoxazole tablets (sulfamethoxazole 0.4 g: trimethoprim 80 mg) were administered immediately after surgery on April 1, 2026, once every 12 h. Twenty-four hours after medication, 75 mg prednisone acetate tablets were added, taken orally in the morning once daily, with a 5 mg dose reduction every three days. Compound sulfamethoxazole tablets were discontinued one week after corticosteroid withdrawal. Adjuvant therapies including gastric protection, potassium supplementation and calcium supplementation were provided.

**Figure 2:**
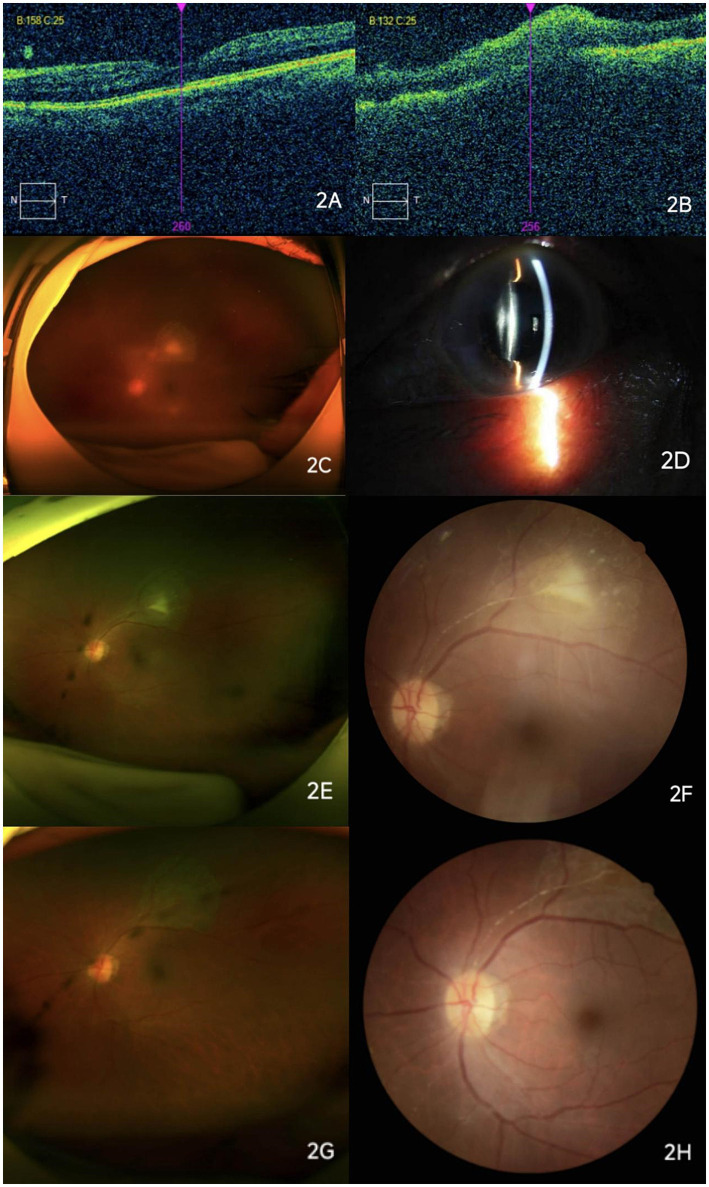
Postoperative images of the affected left eye. **(A)** OCT image of the macula 3 days postoperatively. **(B)** OCT image of the lesion 3 days postoperatively. **(C)** SLO image 3 days postoperatively. **(D)** Anterior segment photograph 3 days postoperatively. **(E)** SLO image 10 days postoperatively. **(F)** 45° fundus photograph 10 days postoperatively. **(G)** SLO image 4 weeks postoperatively. **(H)** 45° fundus photograph 4 weeks postoperatively.

Re-examination three days after surgery: Ophthalmic examination: left eye visual acuity 0.2, intraocular pressure 11 mmHg, conjunctival congestion, sutures in place, transparent cornea, normal anterior chamber depth, aqueous flare, clear iris texture, round pupil with drug-induced mydriasis, well-positioned intraocular lens, flat retina, yellow-white lesion above the macular vascular arch, good laser spot reactions around the lesion, and white linear changes of retinal arteries (Figures 2C,D). Macular OCT revealed an irregular retinal pigment epithelium layer in the macular region, with improvement of macular edema compared with the preoperative findings. OCT through the lesion demonstrated localized retinal thickening and elevation, disorganization of the retinal layers, heterogeneous reflectivity of the outer retina and RPE complex with focal hyperreflective changes, disruption of the ellipsoid zone/external limiting membrane, and underlying signal attenuation (Figures 2A,B). Ten days after treatment, the patient returned to our hospital for re-examination (Figures 2E,F). Ophthalmic examination: left eye visual acuity 0.4, intraocular pressure 14 mmHg, transparent cornea, normal anterior chamber depth, clear aqueous humor, well-positioned intraocular lens, transparent vitreous cavity, flat retina, laser spots surrounding the lesion above the macula, and scarring in the lesion area. Four weeks after treatment, the patient returned to our hospital for re-examination. Fundus examination revealed an attached retina, with white linear or sheathing changes in some retinal arteries. Laser photocoagulation spots were observed around the lesion superior to the macula, and the yellowish-white lesion showed marked regression (Figures 2G,H).

## Discussion

*Toxoplasma gondii* is an obligate intracellular parasite that must enter host cells to survive and proliferate. This characteristic enables it to evade immune clearance and allows the infection to persist in a latent state or undergo recurrent reactivation. Felids serve as its definitive host. Humans are most commonly infected through the ingestion of undercooked meat containing tissue cysts, or through the consumption of water, food, or soil contaminated with oocysts (14, 16, 24). Following primary maternal infection during pregnancy, the pathogen can also be transmitted to the fetus via the placenta (23, 25–28). Therefore, even when a patient denies any history of contact with cats, infection with *Toxoplasma* remains possible. A previous systematic review and meta-analysis reported that the prevalence of ocular toxoplasmosis is approximately 2% in the general population, about 9% among patients with uveitis, and can be as high as 33% in patients with posterior uveitis (18). However, reliable national data on the annual incidence and consultation rate of ocular toxoplasmosis in China are still lacking.

In immunocompetent individuals, *Toxoplasma* infection is mostly asymptomatic, while a minority may present with fever, myalgia, and lymphadenopathy. When the eye is involved, patients commonly present with decreased vision, blurred vision, floaters, photophobia, ocular pain, or eye redness. The typical fundus findings are focal, yellowish-white necrotizing retinitis or retinochoroiditis, often accompanied by vitritis; active lesions may also arise adjacent to old, pigmented chorioretinal scars. Some patients may develop concurrent anterior uveitis, retinal vasculitis, optic nerve involvement, macular edema, or retinal detachment. When the lesion involves the macula or the optic disc, the visual prognosis is often poor.

The diagnosis of ocular toxoplasmosis is primarily based on fundus findings and supplemented by laboratory investigations. For atypical cases, the Goldmann-Witmer coefficient (GWC) is regarded as an important laboratory diagnostic tool. The GWC assesses whether local intraocular antibody production is present by comparing the ratio of *Toxoplasma*-specific IgG to total IgG in intraocular fluid and serum (14, 16). In China, a GWC > 4 is generally considered to support a confirmed diagnosis, while a value between 2 and 4 is suggestive of the disease. The detection of *Toxoplasma* DNA in intraocular fluid by polymerase chain reaction (PCR) also provides direct pathogen evidence. A more prudent clinical approach is to integrate the typical clinical presentation, serological testing, GWC, and PCR results of intraocular fluid. The literature also emphasizes that typical clinical features are crucial for diagnosis, and a positive intraocular fluid PCR result can provide confirmatory evidence. Therefore, although the GWC was <4 in the present case, the diagnosis of left eye ocular toxoplasmosis was still established based on the combination of typical clinical manifestations and a positive intraocular fluid *Toxoplasma* DNA result.

The combination of pyrimethamine, sulfadiazine, and folinic acid, with the addition of corticosteroids when necessary, constitutes the classic “triple therapy” for ocular toxoplasmosis. However, pyrimethamine can cause bone marrow suppression, and sulfadiazine can lead to allergic reactions, gastrointestinal disturbances, hepatorenal toxicity, and hematologic adverse events. Consequently, trimethoprim-sulfamethoxazole or trimethoprim is often used as an alternative in clinical practice, combined with corticosteroids when needed to control inflammation (11, 13, 18, 28, 29). In this case, the patient was treated with trimethoprim-sulfamethoxazole tablets, and corticosteroids were added 24 h later. The postoperative outcome remained stable.

In recent years, diagnostic vitrectomy has gained considerable value in the management of severe vitreous opacity caused by ocular toxoplasmosis. Ocular toxoplasmosis is frequently associated with significant vitritis, and the accumulation of large numbers of inflammatory cells, proteinaceous exudates, and hemorrhage can lead to dense vitreous opacity. This not only causes marked floaters and visual decline but also severely hinders the observation and evaluation of fundus lesions. Vitrectomy should be considered when vitreous opacity persists without significant improvement following standard anti-Toxoplasma and corticosteroid therapy, or when the opacity has already caused a substantial impact on visual function. By removing the opacified vitreous, surgery can immediately restore the transparency of the optical media, significantly improve the patient’s vision, and create the conditions for postoperative fundus monitoring and disease assessment. Intraocular fluid can also be collected simultaneously during the procedure for etiological clarification. However, it must be emphasized that vitreous surgery is only a local therapeutic intervention, and systemic anti-Toxoplasma medication must be continued postoperatively to control the infection and prevent recurrence of inflammation (30–34).

Laser photocoagulation also exerts a certain direct therapeutic and control effect on active toxoplasmic retinal lesions. For localized, active retinitis lesions not adjacent to the macula or optic disc, laser energy can directly destroy the retinal and choroidal tissues parasitized by *Toxoplasma* through its thermal coagulation effect, thereby reducing the pathogen load and promoting the transition of active lesions toward scarring. Simultaneously, photocoagulation can seal the microvessels and choriocapillaris surrounding the lesion, reduce inflammatory exudation and edema, help to limit the spread of local inflammation, and decrease the risk of secondary subretinal fluid accumulation and secondary retinal breaks (33). After the inflammation tends toward quiescence, the firm chorioretinal adhesion formed by the laser scar can serve as a barrier, completely surrounding and isolating the lesion to prevent the spread of inflammation induced by the reactivation of latent tissue cysts. However, the indications for laser treatment during the active phase must be strictly controlled, as excessive energy may exacerbate the inflammatory response or even induce new tissue damage. Therefore, it is advisable to moderately reduce the laser power, aiming for a light gray reaction, so as to avoid excessive photocoagulation. Systemic anti-Toxoplasma medication and corticosteroid therapy must still be combined with laser treatment to more effectively control the lesion and preserve visual function. In the present case, encircling laser photocoagulation was applied around the lesion superior to the macula during surgery. Postoperatively, the lesion showed stable scarring, the inflammation resolved, and no recurrence was observed at the 1-month outpatient follow-up. At the 3-month telephone follow-up, the patient reported no decrease in visual acuity. These findings support the effectiveness of this treatment strategy (21, 33).

Several limitations of this case report should be acknowledged. First, this report describes only one patient, and the follow-up period was relatively short. Although the patient’s visual acuity improved and the retinal lesion showed scarring during follow-up, long-term recurrence could not be fully evaluated. Second, follow-up serological testing for Toxoplasma IgM and IgG was not performed. Therefore, the systemic response to oral therapy and the adequacy of post-treatment monitoring could not be fully assessed. Previous literature reported that 22% of patients developed reactivation between 1.3 and 9 months after 23-gauge pars plana vitrectomy with direct 532 nm endolaser application to and around the active retinitis area. Therefore, the present patient may still have a risk of recurrence (33). Longer follow-up, repeated serological testing, and careful fundus monitoring are needed.

## Data Availability

The raw data supporting the conclusions of this article will be made available by the authors, without undue reservation.
